# Objective adherence to oral appliance therapy in patients with obstructive sleep apnea: a one-year longitudinal analysis

**DOI:** 10.1093/ejo/cjaf037

**Published:** 2025-06-25

**Authors:** Yanlong Chen, Benjamin T Pliska, Bingshuang Zou, Fernanda R Almeida

**Affiliations:** Division of Orthodontics, Department of Oral Health Sciences, Faculty of Dentistry, University of British Columbia, Vancouver, BC V6T 1Z3, Canada; Division of Orthodontics, Department of Oral Health Sciences, Faculty of Dentistry, University of British Columbia, Vancouver, BC V6T 1Z3, Canada; Division of Orthodontics, Department of Oral Health Sciences, Faculty of Dentistry, University of British Columbia, Vancouver, BC V6T 1Z3, Canada; Division of Orthodontics, Department of Oral Health Sciences, Faculty of Dentistry, University of British Columbia, Vancouver, BC V6T 1Z3, Canada

**Keywords:** obstructive sleep apnea, oral appliance therapy, mandibular advancement device, adherence, predictors

## Abstract

**Introduction:**

Oral appliance (OA) therapy is widely used as an alternative to continuous positive airway pressure (CPAP) therapy for treating obstructive sleep apnea (OSA). Traditionally, OA adherence has been assessed through subjective self-reports before, but the availability of objective adherence sensors now allows for more accurate monitoring. This study aimed to analyze one-year objective adherence data to identify adherence patterns over time and factors influencing adherence to OA therapy.

**Materials and Methods:**

Fifty-five OSA patients were recruited from a cohort study and underwent clinical follow-ups at baseline, 1, 6 and 12 months. Patients were treated with custom-made, titratable OAs, and adherence was objectively collected using embedded sensors. Adherence data were analyzed using both intention-to-treat (ITT) and per-protocol (PP) approaches. Statistical methods, including comparative analyses, logistic regression models, and multivariate linear regression were performed to identify predictors of adherence.

**Results:**

Twenty-one patients dropped out before the 12-month follow-up, leaving 34 completed the entire study. At the 1-month follow-up, 80.0% of patients were classified as adherent, with a mean wearing time of 5.98 ± 2.38 hours per night. By 6 months, adherence decreased to 67.3%, with a mean wearing time of 5.69 ± 2.08 hours per night. Several significant predictors of adherence were identified, including larger baseline overjet, younger age, and marital status.

**Conclusions:**

OA adherence declined significantly within the first 6 months but stabilized between 6 and 12 months. Key baseline factors, such as larger overjet, younger age, and being married or partnered are predictors of better adherence, while psychological Comorbidities are associated with lower adherence.

## Introduction

Obstructive sleep apnea (OSA) is a common sleep disorder characterized by periodic and repetitive, partial or complete collapse of the upper airway during sleep, resulting in disrupted sleep patterns and intermittent hypoxemia [1]. This condition is associated with various adverse health outcomes, including cardiovascular disease, stroke, metabolic disorders, and impaired cognitive function [2].

Effective treatments for OSA include medical devices, behavioral interventions, and surgery [1]. Among all, continuous positive airway pressure (CPAP) therapy is widely accepted as the primary therapy for individuals with symptomatic OSA of any severity [3]. However, CPAP is associated with low adherence rates, with approximately 50% of patients discontinuing treatment within the first year [4].

The American Academy of Sleep Medicine (AASM) guidelines recommended the management of OSA patients by oral appliance (OA) to patients who are intolerant to PAP or prefer an alternate therapy [5]. OA therapy, particularly mandibular advancement devices (MADs), effect by repositioning the mandible forward to maintain airway patency during sleep [6]. OA therapy has been proven effective for 50% to 70% of OSA patients, generally with better adherence and tolerance compared to CPAP [7]. Therefore, in terms of treatment effectiveness which is a combination of both efficacy and adherence [8], the end result of OA and CPAP is about the same [9].

Previous studies have reported varying adherence rates to OA therapy, with higher adherence observed in the short term [7], followed by a significant decline in the long term [10]. Most drop-outs happened within the first two years of treatment [11]. Predictors of better OA adherence include using OA as a first-line treatment, experiencing complete symptom resolution, using titratable and custom-made OAs, and receiving supports from bed partners [12].

However, much of the existing adherence data relied on subjective patient self-reports, which are prone to overestimation [8]. Recently, reliable, durable and accurate sensors embedded in OAs have been introduced, enabling the recording of long-term objective adherence data [13]. In this study, we aim to analyze one-year objective adherence data to assess the patterns of OA therapy adherence in both short and long term, and to identify baseline factors that may influence adherence. By understanding these patterns and predictors, we seek to provide evidence that could enhance treatment adherence and aid clinicians in optimizing therapy for individual patients.

## Materials and methods

### Study design

The study was approved by the Clinical Research Ethics Board of The University of British Columbia (Certificate No. H24-02039).

Patients were consecutively recruited from the ORal Appliance Network on Global Effectiveness (ORANGE) cohort [14] at the Faculty of Dentistry, The University of British Columbia between May 2022 and May 2024. All patients meeting the inclusion criteria and not the exclusion criteria were invited to participate. After receiving a full explanation of the study details, patients who provided written informed consent enrolled.

The inclusion criteria were: (i) adults (> 19 years) diagnosed with OSA by overnight sleep study; (ii) patients eligible for OA treatment. The exclusion criteria were: (i) patients who received combined OA and CPAP therapy; (ii) patients who chose OAs without objective adherence sensors. Since this study was exploratory and part of an ongoing cohort, a priori power calculation was not performed for a specific effect size. Instead, we aimed to recruit as many eligible patients as possible over the study period, anticipating a roughly 30%~40% drop-out rate based on previous experience and the settings of COVID-19 pandemic.

Patients attended four clinical visits: at baseline, 1 month, 6 months, and 12 months. At the baseline visit, comprehensive data were collected, including background information (age, gender, education Level, marital status), life habits (smoking, cannabis, alcohol, caffeine consumption), medical history, oral exams (overjet, overbite), and anthropometric measurements (weight, height). Education level was recorded and classified as “higher education” (completion of post-secondary education, including college, university or above) versus “no higher education” (completion of high school or below). Sleep study results, including apnea-hypopnea index (AHI) and oxygen desaturation index (ODI), were retrieved from original reports. Patients also completed questionnaires assessing daytime sleepiness (Epworth Sleepiness Scale, ESS) and quality of life (Functional Outcomes of Sleep Questionnaire, FOSQ-10). At the 1-month, 6-month, and 12-month visits, patients returned for a routine check of the OAs, and the adherence data was downloaded.

### OA therapy

Patients were treated with SomnoDent Flex^®^ (SomnoMed, Leamington, ON, Canada), a bi-bloc, custom-made, and titratable mandibular advancement device. During the baseline visit, the central relation and maximum mandibular protrusion were assessed using the George Gauge (Great Lakes Orthodontics, Tonawanda, NY, USA). OA was initially set at 60% of the maximum protrusion with a standard 5-mm vertical dimension. The titration process began one month after the baseline visit, where patients were instructed to gradually advance the OA to 75% or 90% of maximum protrusion until subjective symptom relief. Subjective symptom relief was defined broadly: if patients continued to experience OSA-related symptoms, the mandible was advanced further. Titration was stopped when the patient reported marked improvement in these symptoms or when side effects (e.g. pain, temporomandibular disorders, chewing problems, or significant bite changes) dictated no further advancement. If side effects became intolerable, the advancement was adjusted backward until improvement.

### Objective adherence data

**Figure 1.**
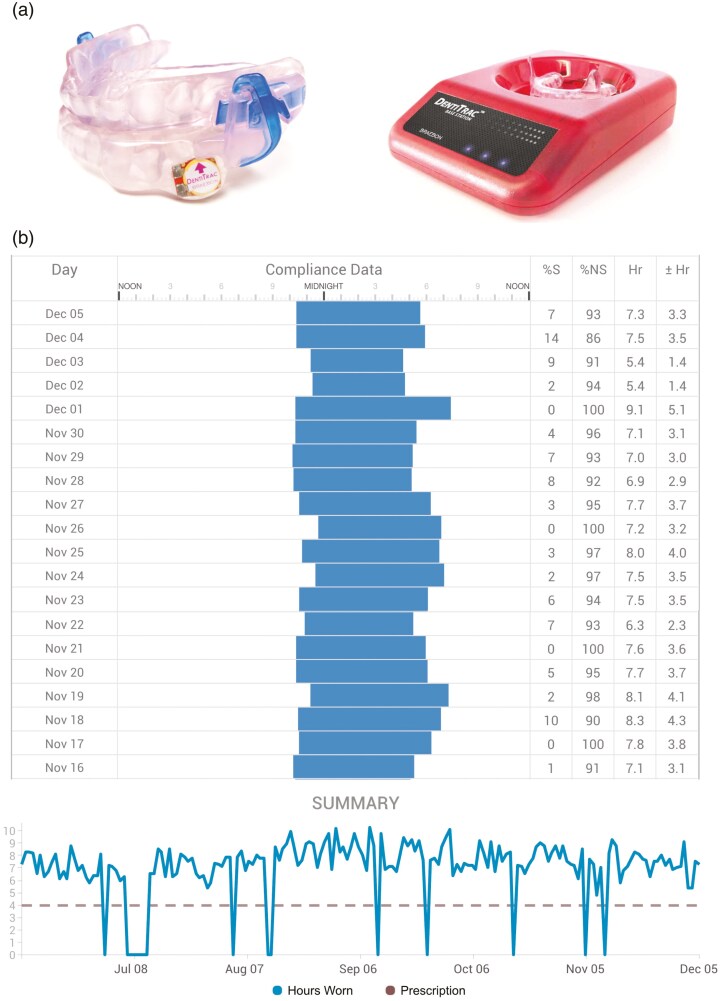
Oral appliance with objective adherence sensor. **(a)** (Left) The DentiTrac^®^ sensor embedded in the lower appliance. (Right) The DentiTrac^®^ base station. **(b)** Example readout from the Braebon^®^ Cloud Service, showing nightly wear duration over 200 days.

Objective adherence data were recorded using the DentiTrac^®^ (Braebon, Kanata, ON, Canada), a thermal sensor embedded into the buccal posterior area of the lower piece of the appliance (**Fig. 1**). The sensor recorded nightly hours when the temperature exceeded 35°C, at a sampling rate of 15 minutes. At each follow-up visit, the OA was placed into a DentiTrac^®^ base station, where the sensor wirelessly transmitted data and uploaded it to a cloud-based system (Braebon Cloud Services, Kanata, ON, Canada).

For each patient, the mean wearing hours per night and the percentage of nights with ≥ 4 hours of use were calculated and referred to as continuous adherence data. Following Pahkala et al. [15], patients were categorized as adherent users if their mean wearing time was ≥ 4 hours per night, and as non-adherent if it was < 4 hours per night. These categorizations were referred to as dichotomous adherence data.

### Statistical analysis

All statistical analyses were performed using IBM SPSS Statistics, Version 26.0 (IBM Corp, Chicago, IL, USA) and JMP^®^, Version 18.0 (SAS Institute Inc., Cary, NC, USA). A two-sided *P* value of < 0.05 was considered statistically significant. The normality of continuous variables was assessed using the Kolmogorov–Smirnov test. Continuous variables were then expressed as mean with standard deviation for normally distributed data or as median with interquartile range for skewed distributions.

To assess the potential impact of drop-outs, baseline characteristics were first compared between patients who completed the 12-month follow-up and those who dropped out. Comparison of continuous variables were conducted using independent Students’ *t*-test for normally distributed data or Mann–Whitney *U*-test for non-normal distributions, while dichotomous variables were analyzed suing Chi-square tests. Both intention-to-treat (ITT) and per-protocol (PP) analyses were conducted: ITT analysis included all available adherence data regardless of completion, while PP analysis included only those who completed the entire study.

To visualize changes in adherence over time, fitting lines were generated from the scatter plots of individual adherence data at 1-month intervals. Differences in wearing hours per night and the percentage of nights with ≥ 4 hours of use at 1, 6, and 12 months were compared using paired *t*-test, while differences in the percentage of adherent users were analyzed using McNemar test.

Logistic regression models were employed to identify factors predicting adherence, adjusting for potential confounders such as age, gender, and BMI. To enhance the model’s predictive power, baseline characteristics were first compared between the adherent and non-adherent users at 1, 6, and 12 months. Variables showing the most statistically significant differences between the groups were manually entered into the regression model.

## Results

### Patient characteristics

As shown in **Table 1**, a total of 55 patients (35 males and 20 females) with a mean age of 54.3 ± 11.4 years and a mean BMI of 27.35 ± 4.41 kg/m^2^ were initially recruited for the study. The majority of patients were diagnosed with moderate to severe OSA, with a mean AHI of 16.20 ± 14.30 events/hour. The patients were relatively well-educated, with approximately 85% having completed at least post-secondary education. Alcohol consumption was reported by 69.8% of patients, while 77.4% consumed caffeine regularly. Most patients exhibited normal levels of daytime sleepiness, with a mean ESS score 7.60 ± 5.30.

**Table 1.** Patient characteristics.

Baseline Characteristics	Initially recruited (N = 55)	Completed 12m follow-up (N = 34)	Drop-outs before 6m (N = 6)	*P*_1_	Drop-outs between 6m and 12m (N = 15)	*P*_2_
Age, y, mean (SD)	54.3 (11.4)	55.1 (9.7)	59.0 (11.9)	.476	50.53 (14.07)	.268
Gender, Male, N (%)	35 (63.6)	23 (67.6)	5 (83.3)	.440	7 (46.7)	.165
BMI, kg/m^2^, mean (SD)	27.35 (4.41)	27.36 (4.78)	26.94 (3.68)	.825	27.47 (3.89)	.935
AHI, events/h, mean (SD)	16.20 (14.30)	16.54 (12.91)	27.70 (23.47)	.303	10.42 (10.07)	.120
Education, Higher, N (%)	45 (84.9)	31(91.2)	4 (67.7)	.442	10 (66.7)	.078
Marriage, Married or partnered, N (%)	41 (75.9)	23 (67.6)	3 (50.0)	.735	11 (73.3)	.449
Smoking, Current, N (%)	4 (7.4)	3 (8.8)	1 (16.7)	.496	0 (0)	.431
Cannabis, Current, N (%)	16 (30.8)	11 (32.4)	0 (0)	.133	5 (33.3)	.693
Alcohol, Current, N (%)	37 (69.8)	23 (67.6)	3 (50.0)	.735	11 (73.3)	.449
Caffeine, Current, N (%)	41 (77.4)	26 (76.5)	4 (67.7)	.861	11 (73.3)	.875
Overjet (mm), mean (SD)	3.45 (2.78)	3.50 (2.78)	2.92 (2.01)	.554	3.57 (3.17)	.944
Overbite (%), mean (SD)	34.00 (26.38)	33.68 (24.93)	30.00 (18.97)	.688	36.33 (32.81)	.782
ESS Score, mean (SD)	7.60 (5.30)	6.44 (4.49)	13.60 (8.65)	.138	8.31 (4.42)	.211
FOSQ-10, mean (SD)	16.01 (3.71)	17.10 (7.91)	10.48 (4.63)	.131	15.23 (3.35)	.104

*P*_1_, the *P* value between completed and drop-outs before 6 months; *P*_2_, the *P* value between completed and drop-outs between 6 and 12 months. AHI, apnea-hypopnea index; BMI, body mass index; ESS, Epworth Sleep Scale; FOSQ-10, Functional Outcomes of Sleep Questionnaire-10; SD, standard deviation.

^*^ *P* < 0.05 was considered statistically significant.

All the 55 patients had attended the 1-month follow-up. However, 6 patients dropped out before the 6-month follow-up, and an additional 15 dropped out between 6 and 12 months, leaving 34 patients who completed the entire study. This study, launched during the COVID-19 pandemic, faced challenges with 12 of the 21 drop-outs attributed to lockdowns or commuting difficulties, 4 due to health or safety concerns, and 5 due to unsatisfactory treatment effects or significant side effects. As shown in Table 1, no statistically significant differences were observed in baseline characteristics between patients who completed the study and those who dropped out, suggesting that the impact of drop-outs on the study results was likely not significant.

### Adherence change over time

**Figure 2.**
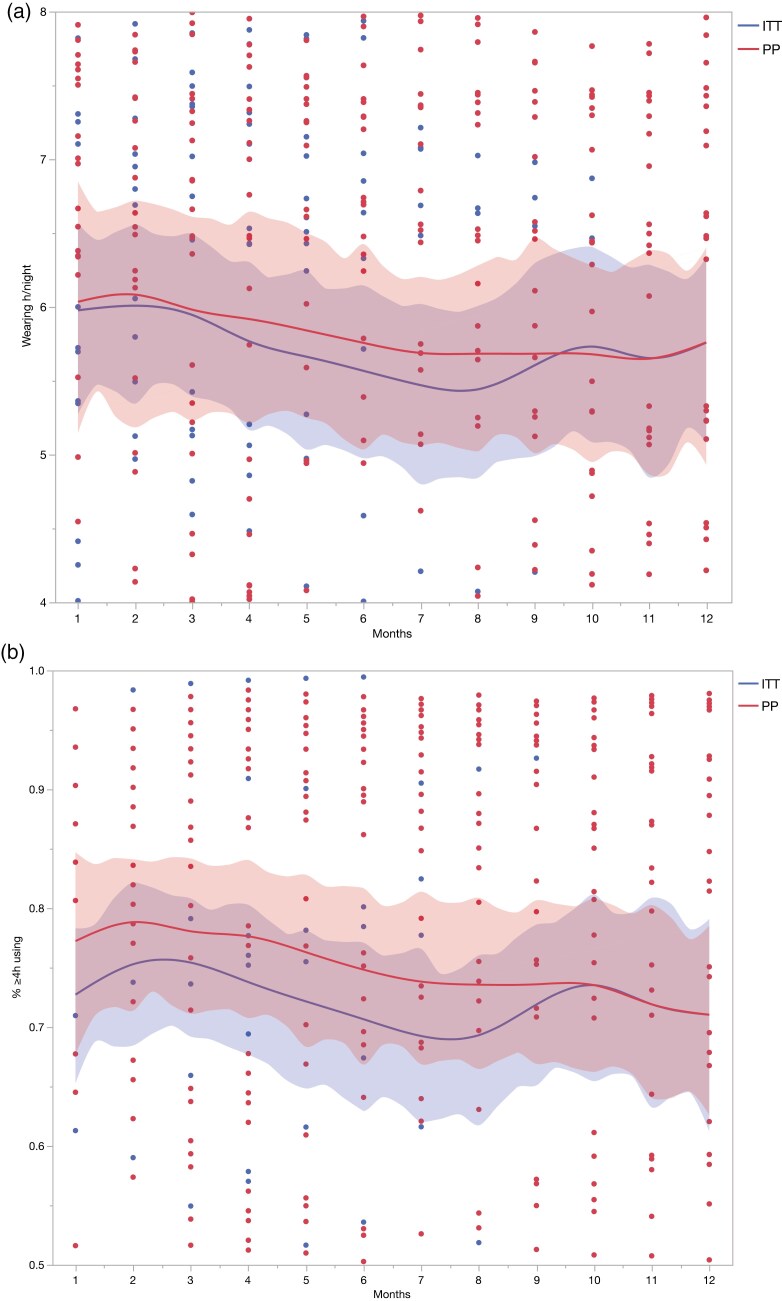
Objective oral appliance adherence changes over time. **(a)** Mean wearing time (hours/night). **(b)** Percentage of nights with ≥ 4 hours use. Fitting lines were generated from the scatter plots of individual adherence data at the interval of 1 month. Shading areas represent 90% confidence interval. OA, oral appliance; ITT, intention-to-treat analysis; PP, per-protocol analysis.

As shown in **Fig. 2** and **Table 2**, both ITT and PP analyses showed that OA adherence decreased sharply during the first 6 months of treatment, and then remained relatively stable from 6 to 12 months. During the first 6 months, adherence in the ITT analysis were slightly lower than in the PP analysis, likely due to initial drop-outs related to patient dissatisfaction or short-term side effects. Both analyses showed a tendency that males generally had better adherence than females across all characteristics analyzed.

**Table 2.** Objective adherence patterns at 1, 6, and 12-month follow-up.

Adherence characteristics	1 month	6 months	*P*_1_	12 months	*P*_2_	*P*_3_
Intention-to-treat analysis						
N (male/female)	55 (35/20)	49 (30/19)		34 (23/11)		
Wearing hours / night (h), Mean (SD)						
Total	5.98 (2.38)	5.69 (2.08)	0.261	5.82 (2.06)	0.389	0.379
Male	6.07 (2.25)	5.80 (2.16)	0.313	6.07 (1.97)	0.394	0.255
Female	5.81 (2.64)	5.52 (2.01)	0.352	5.30 (2.23)	0.296	0.391
Percentage of ≥ 4 hours using (%), Mean (SD)						
Total	74.42 (28.93)	70.98 (24.28)	0.258	73.03 (23.68)	0.35	0.409
Male	76.91 (26.68)	73.17 (24.58)	0.28	76.37 (22.17)	0.322	0.312
Female	70.05 (32.77)	67.53 (24.02)	0.393	66.00 (26.29)	0.364	0.436
Adherent users, N (%)						
Total	44 (80.0)	33 (67.3)	0.092	28 (82.4)	0.898	0.063
Male	30 (85.7)	19 (63.3)	.039^*^	19 (82.6)	0.248	0.25
Female	14 (70.0)	14 (73.4)	0.999	9 (81.8)	0.48	0.5
Per-protocol analysis						
N (male/female)	34 (23/11)	34 (23/11)		34 (23/11)		
Wearing hours / night (h), Mean (SD)						
Total	6.23 (2.37)	5.97 (2.01)	0.224	5.82 (2.06)	0.152	0.132
Male	6.43 (2.14)	6.24 (1.96)	0.28	6.07 (1.97)	0.128	0.197
Female	5.79 (2.87)	5.43 (2.11)	0.32	5.30 (2.24)	0.344	0.289
Percentage of ≥ 4 hours using (%), Mean (SD)						
Total	78.73 (28.05)	74.90 (22.84)	0.176	73.03 (23.68)	0.15	0.127
Male	81.74 (24.45)	78.38 (21.27)	0.208	76.37 (22.17)	0.156	0.161
Female	72.42 (34.87)	67.63 (25.30)	0.313	66.06 (26.28)	0.346	0.284
Adherent users, N (%)						
Total	28 (82.4)	23 (67.6)	0.18	28 (82.4)	0.063	0.999
Male	20 (87.0)	16 (69.6)	0.219	19 (82.6)	0.25	0.999
Female	8 (72.7)	7 (63.6)	0.999	9 (81.8)	0.5	0.999

*P*_1_, the *P* value between the 1-month and 6-month group. *P*_2_, the *P* value between the 1-month and 12-month group. *P*_3_, the *P* value between the 6-month and 12-month group. SD, standard deviation.

^*^ *P* < 0.05 was considered statistically significant.

In the ITT analysis, 80.0% of patients were classified as adherent users at the 1-month follow-up, with a mean wearing time of 5.98 ± 2.38 hours per night and 74.42 ± 28.93% of nights with ≥ 4 hours of use. By the 6-month follow-up, adherence rates had decreased to 67.3%, with the mean wearing time dropping to 5.69 ± 2.08 hours per night and the percentage of nights with ≥ 4 hours of use falling to 70.98 ± 24.28%. By the 12-month follow-up, the adherence rate had slightly increased to 82.4%, with a mean wearing time of 5.82 ± 2.06 hours per night and 73.03 ± 23.68% of nights with ≥ 4 hours of use, although these changes were not statistically significant.

In the PP analysis, the mean wearing time decreased from 6.23 ± 2.37 hours at 1month to 5.97 ± 2.01 hours at 6 months, and then to 5.82 ± 2.06 hours by the 12-month follow-up. The percentage of nights with ≥ 4 hours of use also declined, from 78.73 ± 28.05% at 1 month to 74.90 ± 22.84% at 6 months, and then to 73.03 ± 23.68% at 12 months. The adherence rate remained 82.4% at both the 1-month and 12-month follow-ups, with a dip to 67.6% at 6 months. No variables in the PP analysis showed statistical significance.

### Characteristics of adherent versus non-adherent users and prediction models

Comparative analyses between adherent and non-adherent users were presented in **Table 3**. At the 1-month follow-up, adherent users had significantly larger overjet compared to non-adherent users (3.80 ± 2.90 mm versus 2.09 ± 1.74 mm, *P* = 0.019). Additionally, adherent users were more likely to be married or partnered, while non-adherent users tended to be single, divorced, or widowed (OR: 9.028, *P* = 0.060). At the 6-month follow-up, adherent users were significant older than non-adherent users (56.12 ± 10.37 years versus 48.69 ± 11.77 years, *P* = 0.040) and also more likely to be married or partnered (OR: 11.653, *P* = 0.020). By the 12-month follow-up, age was the only significant factor distinguishing adherent from non-adherent users, with adherent users tending to be older (56.61 ± 8.90 years versus 48.00 ± 11.13 years, *P* = 0.048).

**Table 3.** Comparative analyses between adherent and non-adherent users at 1, 6, and 12-month follow-up.

Baseline characteristics	1 month (N = 55)				6 months (N = 49)				12 months (N = 34)			
	Adherent (N = 44)	Non-adherent (N = 11)	Odds Ratio	*P*_1_	Adherent (N = 33)	Non-adherent (N = 16)	Odds Ratio	*P*_2_	Adherent (N = 28)	Non-adherent (N = 6)	Odds Ratio	*P*_3_
Age (years), mean (SD)	54.45 (10.96)	53.55 (13.43)		.838	56.12 (10.37)	48.69 (11.77)		.040^*^	56.61 (8.90)	48.00 (11.13)		.048^*^
Gender, male, n (%)	30 (54.5)	5 (9.1)	1.964	.161	19 (38.8)	11 (24.4)	0.567	.452	19 (55.9)	4 (11.8)	0.003	.955
BMI (kg/m^2^), mean (SD)	27.11 (4.57)	28.31 (3.73)		.397	27.99 (3.43)	25.99 (6.32)		.280	27.19 (5.07)	28.13 (3.34)		.670
AHI (events/h), mean (SD)	14.63 (12.22)	22.31 (20.11)		.250	16.33 (12.64)	11.61 (11.53)		.205	17.46 (13.77)	12.28 (6.99)		.381
Education, higher education, n (%)	35 (63.6)	10 (18.2)	0.390	.532	28 (57.1)	13 (26.5)	0.027	.869	25 (73.5)	6 (17.6)	0.705	.401
Marriage, n (%)												
Single/Divorced/Widowed	9 (16.4)	6 (10.9)			3 (6.1)	8 (16.3)			23 (67.6)	3 (8.8)		
Married/Partnered	35 (63.6)	5 (9.1)	9.028	.060	30 (61.2)	8 (16.3)	11.653	.020^*^	5 (14.7)	3 (8.8)	5.616	.230
Smoking, n (%)			0.057	.972							1.050	.591
Never	24 (43.6)	6 (10.9)			17 (34.7)	9 (18.4)	0.358	.836	15 (44.1)	2 (5.9)		
Past	16 (29.1)	4 (7.3)			14 (28.6)	5 (10.2)			11 (32.4)	3 (8.8)		
Current	3 (5.5)	1 (1.8)			2 (4.1)	2 (4.1)			2 (5.9)	1 (2.9)		
Cannabis, n (%)	11 (20.0)	5 (9.1)	1.412	.235	11 (22.4)	5 (10.2)	0.025	.875	9 (26.5)	2 (5.9)	0.003	.955
Alcohol, n (%)	30 (54.5)	7 (12.7)	0.251	.616	26 (53.1)	8 (16.3)	3.234	.072	20 (58.8)	3 (8.8)	1.037	.309
Caffeine, n (%)	32 (58.2)	9 (16.4)	0.158	.691	26 (53.1)	11 (22.4)	0.174	.677	22 (64.7)	4 (11.8)	0.389	.533
Medical condition, n (%)												
Hypertension	11 (20.0)	5 (9.1)	1.535	.215	7 (14.3)	5 (10.2)	0.808	.369	9 (26.5)	1 (2.9)	0.570	.450
Cardiovascular disease	6 (10.9)	0 (0)		.183	4 (8.2)	1 (2.0)	0.329	.566	4 (11.8)	0 (0)	0.971	.324
Depression/Anxiety	15 (27.3)	7 (12.7)	2.799	.094	11 (22.4)	8 (16.3)	1.725	.189`	9 (26.5)	4 (11.8)	2.494	.114
Bruxism	23 (41.8)	6 (10.9)	0.018	.893	18 (36.7)	8 (16.3)	0.089	.765	15 (44.1)	3 (8.8)	0.025	.874
Overjet (mm), mean (SD)	3.80 (2.90)	2.09 (1.74)		.019^*^	3.20 (2.00)	4.19 (4.13)		.375	3.43 (2.57)	3.83 (3.87)		.751
Overbite (%), mean (SD)	35.68 (26.09)	27.27 (27.69)		.377	35.00 (27.16)	33.44 (28.33)		.856	33.04 (24.13)	36.67 (30.77)		.752
ESS Score, mean (SD)	7.90 (4.98)	6.45 (6.50)		.504	7.31 (4.78)	6.20 (3.90)		.403	6.75 (4.60)	5.00 (4.00)		.395
FOSQ-10, mean (SD)	16.24 (3.29)	15.18 (5.07)		.523	16.88 (3.43)	16.07 (2.30)		.353	16.96 (3.14)	17.75 (1.44)		.556

Only selected medical conditions with prevalence > 10% are shown. AHI, apnea-hypopnea index; BMI, body mass index; ESS, Epworth Sleep Scale; FOSQ-10, Functional Outcomes of Sleep Questionnaire-10; SD, standard deviation.

^*^ *P* < 0.05 was considered statistically significant.

Logistic regression analyses adjusted by age and gender, where adherence was treated as a binary dependent variable, identified several significant baseline predictors at 1, 6, and 12 months (**Table 4**). At the 1-month follow-up, baseline overjet was a significant predictor, with patients having a larger overjet being more likely to adhere to OA therapy (OR: 1.663, 95% CI: 1.117-2.477, *P* = 0.012). At the 6-month follow-up, adherence at 1 month emerged as a significant predictor, indicating that patients who were initially adherent were more likely to remain adherent (OR: 13.193, 95% CI: 1.580-110.149, *P* = 0.016). By the 12-month follow-up, being married or partnered at baseline was positively associated with adherence (OR: 7.295, 95% CI: 1.044-50.968, *P* < 0.045).

**Table 4.** Binary logistic regression models predicting objective adherence at 1, 6, and 12-month follow-up.

Baseline Characteristics	Odds Ratio	95% Confidence Interval	*P*
**Model 1: 1-month follow-up**			
Age	1.022	[0.945, 1.105]	.586
Gender	1.969	[0.367, 10.567]	.429
Marriage	1.074	[0.362, 3.188]	.898
Psychological diseases	0.386	[0.074, 2.000]	.256
Overjet	1.663	[1.117, 2.477]	.012^*^
**Model 2: 6-month follow-up**			
Age	1.069	[1.000, 1.142]	.072
Gender	0.822	[0.177, 3.823]	.188
Marriage	1.212	[0.476, 3.085]	.275
Alcohol	2.909	[0.627, 13.494]	.081
Psychological diseases	0.326	[0.080, 1.337]	.260
1-month adherence	13.193	[1.580, 110.149]	.016^*^
**Model 3: 12-month follow-up**			
Age	1.055	[0.941, 1.184]	.340
Gender	0.325	[0.020, 5.325]	.431
Marriage	7.295	[1.044, 50.968]	.045^*^
Psychological diseases	0.265	[0.034, 2.041]	.202
1 month adherence	0.304	[0.006, 15.205]	.551

^*^ *P* < 0.05 was considered statistically significant.

In the multivariate linear regression analyses (**Table 5**), psychological diseases were the only variable negatively associated with the percentage of nights with ≥ 4 hours of OA use at both the 6 and 12-month follow-ups. After controlling for the other variables in these models, phycological diseases were associated with an average decrease of 0.411% (R^2^ = 0.196, *P* = 0.016) in nights with ≥ 4 hours of OA use at the 6-month follow-up and 0.435% (R^2^ = 0.264, *P* = 0.019) at the 12-month follow-up.

**Table 5.** Multivariate linear regression models predicting objective adherence at 1, 6, and 12-month follow-up.

Variables	Model 1 (mean wearing time)		Model 2 (nights% of ≥ 4 hours use)	
	β	*P*_*1*_	β	*P*_*2*_
**1-month follow-up**				
Age	0.072	0.599	0.082	0.551
Gender	0.048	0.747	0.087	0.557
Marriage	0.216	0.124	0.148	0.289
Psychological diseases	-0.094	0.529	-0.145	0.331
Overjet	0.114	0.425	0.112	0.434
		**R** ^**2**^ ** = 0.164**		**R** ^**2**^ ** = 0.166**
**6-month follow-up**				
Age	0.098	0.521	0.129	0.381
Gender	-0.012	0.940	0.007	0.964
Marriage	0.052	0.740	0.043	0.996
Alcohol	0.077	0.631	-0.028	0.853
Psychological diseases	-0.286	0.100	-0.411	0.016^*^
		**R** ^**2**^ ** = 0.130**		**R** ^**2**^ ** = 0.196**
**12-month follow-up**				
Age	0.038	0.834	0.104	0.545
Gender	0.083	0.656	0.058	0.736
Marriage	0.194	0.283	0.169	0.317
Psychological diseases	-0.311	0.109	-0.435	0.019^*^
		**R** ^**2**^ ** = 0.157**		**R** ^**2**^ ** = 0.264**

All multivariate linear regression models were adjusted by gender and age.

^*^ *P* < 0.05 was considered statistically significant.

## Discussion

This study analyzed OA adherence patterns over a one-year period and identified several factors that predict adherence. In terms of the adherence patterns, our findings are consistent with previous studies [8, 11, 15], which has shown that while initial adherence is high, there is a significant decline within the first six months, after which adherence tends to stabilize.

Our study also revealed additional novel factors that could affect OA adherence. Firstly, a larger baseline overjet could be a significant predictor of better early adherence, which may be a new insight into how dental and craniofacial factors impact early OA acceptance. Secondly, our study is among the first to demonstrate that psychological comorbidities (e.g. depression or anxiety) can adversely affect long-term OA use, an area where evidence was limited [12]. Moreover, the protective effect of specific sociodemographic factors (e.g. being married or partnered) on adherence was evident at 12 months, highlighting the long-term role and importance of social support of OSA patients.

### Adherence decline and stabilization

The decline in adherence observed during the first 6 months may be attributed to short-term side effects, such as dental pain, muscular pain, and excessive salivation. Previous studies have identified these issues [16], along with a decrease in treatment efficacy, as common reasons for early discontinuation within the first two years of therapy [16, 17]. Interestingly, in our study, adherence rates stabilized between 6 and 12 months. While long-term side effects, such as bite changes, can emerge and potentially affect adherence, these effects were mostly minimal and not prominent within the first year [17–19]. Therefore, it can be inferred that once patients overcome the initial adaptation period, they are more likely to maintain consistent use. This finding highlights the importance of targeted patient support and regular follow-ups during the early months of therapy, which is a critical period for ensuring long-term adherence.

### Predictors of adherence

This study identified several baseline factors associated with better adherence to OA therapy. Firstly, we found that a larger baseline overjet was associated with better adherence at 1 month. This could be because a larger overjet generally allows for more space for mandibular advancement [20], which improves the fit and comfort of the device and makes it more tolerable. However, this advantage appears to be short-term. By 6 months, the overjet difference between adherent and non-adherent patients tended to have a reversed direction. One possible explanation is that there is a dose-dependent effect of mandibular advancement on airway patency. Those with a larger overjet often require a larger mandibular protrusion to effectively treat OSA, which can introduce more discomfort as titration progresses. In other words, while a larger overjet provides space that might improve the comfort and fit initially, the greater degree of protrusion needed might offset that early comfort in the long-term.

Secondly, being married or partnered consistently emerged as a positive predictor of long-term adherence. This finding aligns with previous research showing that social support plays a crucial role in maintaining treatment adherence, as partners often encourage consistent use [21]. Improved bed partner satisfaction level and a significant reduction in socially disturbing snoring further promote consistent OA use [18].

Age also played a significant role, although the relationship between age and adherence was complex.

We think age-related behavioral factors might explain the observed patterns of adherence. Older patients tended to maintain better long-term adherence, which could be due to higher motivation or urgency to address health concerns. They may be more concerned about the consequences of untreated OSA and thus more committed to using the therapy consistently. Older patients might also have more stable schedules that facilitate regular use. In contrast, younger patients may feel a lower immediate risk from OSA symptoms and therefore be less motivated. They might also have busier or more irregular schedules, making it harder to use the oral appliance every night. However, in our study, age was not a strong or consistent predictor of OA adherence and should not be overestimated. Although older patients showed slightly better adherence in the long term, the influence of age was modest, which is in line with other studies reporting only a weak association between patient age and therapy adherence [11, 18, 22, 23].

Our results indicated that patients with psychological comorbidities, such as depression or anxiety, had significantly lower adherence to OA therapy. Patients with depression may have reduced motivation and energy to use the device nightly, while those with anxiety might experience heightened discomfort or worry with the appliance in place, leading to intermittent use [24, 25]. This finding underscores the importance of addressing mental health issues in the management of OSA [26].

### Possible impact of drop-outs

This study experienced a notable dropout rate, with 21 out of 55 patients discontinuing before the 12-month follow-up. These drop-outs could potentially introduce bias into the study results, particularly if the reasons for discontinuation were related to the effectiveness or tolerability of the OA. However, although our recruitment began after the peak of the COVID-19 pandemic, the pandemic’s lingering effects still contributed to the drop-out rate. Many of the 21 drop-outs were due to pandemic-related factors such as travel difficulties and personal health-safety concerns, with only a small number attributed to dissatisfaction with the treatment or significant side effects. Importantly, our comparison of baseline characteristics between those who completed the study and those who dropped out revealed no statistically significant differences. These observations suggest that the impact of drop-outs on the findings may be limited. Nonetheless, the possibility of selection bias cannot be entirely ruled out, and this should be considered when interpreting the results.

### Strengths and limitations

A key strength of this study was the use of objective adherence data, providing a more accurate measure of actual use compared to self-reported data [27]. Previous research has shown that self-reported adherence often overestimates actual use. For example, a randomized controlled trial found a slight overestimation in self-reports (7.3 hours per night subjectively versus 6.6 hours per night objectively) [28], while another study reported a mean overestimation of 30 minutes at the 1-year follow-up [8]. By using objective data, our study provides a clearer assessment of adherence patterns, allowing for a more precise identification of factors that influence both short and long-term OA adherence. Furthermore, the study sample was consecutively recruited from a cohort at our center during the study period and not selectively filtered beyond standard eligibility for OA treatment, which enhances the real-world applicability of our results.

However, this study also has some limitations. Firstly, the sample size was relatively small, and the drop-out rate was higher than anticipated, which may impact the robustness of the findings. These factors should be considered when interpreting results. Secondly, there was considerable variability in baseline characteristics, such as age, BMI, and AHI, which could affect the generalizability of the findings. The follow-up period was relatively short, particularly for assessing long-term adherence. Thirdly, the use of objective sensors and the study design may have influenced patient behavior, as some patients increased their wearing time abruptly before follow-up visits, likely due to awareness that their adherence data would soon be collected. Furthermore, patients who did not experience satisfied symptom improvement might be more likely to discontinue treatment. Since treatment efficacy (e.g. AHI) was not measured in our study, some drop-outs could be related to perceived efficacy of therapy, which could be pursued in future studies.

### Clinical implications

The findings from this study have several implications for clinical practice. First, early intervention strategies, such as enhanced patient education, regular follow-ups, and personalized adjustments to the OA during the initial months of therapy, are crucial for both short and long-term adherence. Although evidence regarding the impact of psychological factors on OA adherence is limited [12], literature on CPAP adherence has shown that interventions on patient education, motivational enhancement, and augmented support can significantly improve adherence compared to standard care [29]. Similarly in OA therapy, we recommended a multidisciplinary and supportive approach to improve adherence in patients with low mood or anxiety. The interventions may include enhanced educational and counseling sessions about OSA and OA therapy, regular motivational follow-ups, and involvement of mental health professionals when appropriate. We also emphasize enhanced social support through partners or through individualized support programs [30].

This study also demonstrates the feasibility and validity of using embedded objective adherence sensors for long-term monitoring. The devices could provide objective data that aligns with expected adherence trends and may reveal details (such as nightly use patterns and adherence patterns changes over time) that could be missed or misestimated by self-report [8]. Thus, integrating such objective sensors into OAs for OSA treatment can greatly enhance accuracy in monitoring patient adherence.

## Conclusions

Based on the findings of this study, the following conclusions were drawn:

1) OA adherence dropped down sharply in the first 6 months, and then remained relatively stable from 6 to 12 months;
2) The 1-month adherence were the only independent predictors of the 6 months adherence;
3) Age, marital status, and psychological diseases might affect both the short-term and long-term adherence. Younger, single, or unaccompanied patients, and patients with psychological diseases tended to have poorer adherence.

## Data Availability

The data underlying this article will be shared on reasonable request to the corresponding author.
